# A Multihop Key Agreement Scheme for Wireless Ad Hoc Networks Based on Channel Characteristics

**DOI:** 10.1155/2013/935604

**Published:** 2013-05-20

**Authors:** Zhuo Hao, Sheng Zhong, Nenghai Yu

**Affiliations:** ^1^MicroStrategy, Hangzhou, China; ^2^State Key Laboratory of Novel Software Technology, Nanjing University, Nanjing 210023, China; ^3^Department of Computer Science and Technology, Nanjing University, Nanjing 210023, China; ^4^Department of Electronic Engineering and Information Science, University of Science and Technology of China, Hefei, Anhui 230027, China

## Abstract

A number of key agreement schemes based on wireless channel characteristics have been proposed recently. However, previous key agreement schemes require that two nodes which need to agree on a key are within the communication range of each other. Hence, they are not suitable for multihop wireless networks, in which nodes do not always have direct connections with each other. In this paper, we first propose a basic multihop key agreement scheme for wireless ad hoc networks. The proposed basic scheme is resistant to external eavesdroppers. Nevertheless, this basic scheme is not secure when there exist internal eavesdroppers or Man-in-the-Middle (MITM) adversaries. In order to cope with these adversaries, we propose an improved multihop key agreement scheme. We show that the improved scheme is secure against internal eavesdroppers and MITM adversaries in a single path. Both performance analysis and simulation results demonstrate that the improved scheme is efficient. Consequently, the improved key agreement scheme is suitable for multihop wireless ad hoc networks.

## 1. Introduction

 Network security (see, e.g., [1, 2]) has been studied extensively. In wireless networks, security problems are especially critical, because wireless channels are inherently broadcast channels. When a pair of nodes communicate with each other, nearby nodes within the communication range may be able to overhear their messages. In order to prevent eavesdropping, messages are often encrypted before being sent. Hence, key agreement is of great importance for security of wireless networks.

Recently, Mathur et al. [3] propose a novel key agreement scheme for wireless networks, which is based on the secrecy of the wireless channel itself. In their scheme, the two communicating nodes send probe signals to each other and measure the channels. Then, they extract secret bits from the channel measurements using a level-crossing algorithm. Because of the reciprocity of the channel, the two nodes can extract the same key from their own channel measurements. Any eavesdroppers that are more than half a wavelength away from both nodes can get no knowledge of the key, because their experienced channels are independent of the channel between the two communicating nodes. The broad applicability of this security alternative has been validated by Jana et al. [4], through a series of experiments in real environments.

However, both Mathur et al.'s and Jana et al.'s schemes require that two nodes are within the communication range of each other in order to establish a key. This requirement cannot always be satisfied. In many realistic scenarios, intermediate nodes are needed for relaying messages, because the end nodes cannot communicate directly.

In this paper, we show that it is feasible to build key agreement schemes based on wireless channel measurements in *multihop* wireless networks. We show that, by extracting secrets from the phase characteristics (it is feasible to extract secrets from phase characteristics—please see Section 3 for details) of channels, two end nodes that are more than one hop away from each other can establish a key between them. We propose a basic key agreement scheme for this purpose and show that it is secure against external eavesdroppers (i.e., eavesdroppers out of the paths connecting the two nodes). After that, we show that the basic scheme is subject to internal eavesdropping and Man-in-the-Middle (MITM) attacks. Therefore, we propose an improved key agreement scheme to prevent these two attacks. The improved scheme is based on the assumption that the network is biconnected. The secrets are extracted from two disjoint paths between the two end nodes. The improved scheme is secure against internal eavesdroppers and MITM adversaries in a single path. (Please see Section 5.3, Remark 7 for the possibility that adversaries control more than a single path.) In both the basic and the improved schemes, we follow the standard assumption [3–6] that adversaries are more than half a wavelength away from all the participating nodes. We give a theoretical analysis of the key agreement probability and show that it is affected by communication SNRs, sampling rates, and quantization parameters. We simulate the improved scheme in GlomoSim [7] and show that the established key has strong randomness and the key agreement efficiency is high.

In summary, we have the following contributions.

- We propose a basic multihop key agreement scheme and prove that it is secure against external eavesdroppers.
- Since the basic scheme is not secure against internal eavesdroppers or MITM adversaries, we propose an improved multihop key agreement scheme, and prove that this improved scheme is secure against internal eavesdroppers and MITM adversaries in a single path between the two nodes.
- We give both performance analysis and simulation results of the improved scheme. The results show that the improved scheme is very efficient and the established key has strong randomness.

The rest of this paper is organized as follows. In Section 2, we review the related work. In Section 3, we present technical preliminaries. In Section 4, we present the basic multihop key agreement scheme and give a security analysis. In Section 5, we describe the improved multihop key agreement scheme and prove its security. In Sections 6 and 7, we show that the improved scheme is efficient by both theoretical analysis and simulation results. Finally, we conclude in Section 8.

## 2. Related Work

 Key agreement based on channel characteristics is firstly proposed in Hershey et al. [8], in which the secret key is extracted from the phase differences of continuous waves. After that, Hassan et al. [9] propose to use phase differences between two orthogonal subcarriers as extracted secrets. Tope and McEachen [10] propose a key generation scheme based on polarity of power envelope differences. Recently, a lot of schemes [3–6, 11–22] are proposed to enhance the security and/or improve the performance. In particular, Mathur et al. [3] propose a scheme to extract secret bits from wireless channel measurements. They design a level-crossing algorithm to increase the bit consistency rate. They do experiments using both customized 802.11 platform and off-the-shelf 802.11 network cards. In order to validate the effectiveness of the key extraction schemes based on signal strengths, Jana et al. [4] carry out extensive experiments in various environments. They propose adaptive quantization method to improve the performance. Patwari et al. [6] propose a high-rate uncorrelated bit extraction scheme based on fractional interpolation, decorrelation transformation and multibit adaptive quantization. Ye et al. [5] propose a secret key extraction approach that is suited for more general channel state distributions. Zhang et al. [21] find that mobility patterns have important impact on the correlation of channel measurements at the end nodes. They show that more diffusion in the mobility brings less correlation in the measured channel impulse responses. Gollakota and Katabi [23] propose a secret communication method based on receiver's jamming. Their method eliminates the reliance on channel variance and has high secret communication speed.

There are also many analytical works [24–27] that provide theoretical analysis of secret key exchange protocols and propose improved algorithms. In addition, secret key extraction schemes from UWB (Ultra-WideBand) channels are proposed in [28–31]. Croft et al. [32] propose a secret bit extraction scheme for wireless sensors, while Ali et al. [33] develop a key extraction approach in body area networks.

It is important to note that all the previous approaches focus on one single channel between two nodes. Therefore, they have the requirement that the two nodes are within the communication range of each other. In contrast, in this paper, we propose schemes that are suitable for multihop networks, in which nodes can be out of the communication range of each other. Consequently, our proposed schemes can be used for key agreement in multihop wireless networks.

Recently Wang et al. [34] propose a group key agreement scheme in wireless networks. Wang et al.'s scheme is based on the phase characteristics of wireless channels. They use phase randomness for bit generation and remove the reliance on the node mobility. According to Ren et al. [35], phase-based methods [8, 9, 16, 34] have three advantages compared to RSS-based methods [3–6], including having uniform distribution, providing high resolution phase estimation, and enabling phase accumulation across multiple nodes. Similar to [34], the schemes proposed in this paper are also based on channel phase randomness. However, our proposed schemes consider a completely different setting, in which the involved nodes can be more than one hop away from each other. In fact, allowing nodes to be multiple hops away from each other is a major technical challenge addressed in this paper. Hence, our schemes are independent from, and complementary to, the results in [34].

## 3. Technical Preliminaries

 In a typical multihop mobile ad hoc network, there are no infrastructures. Each node is both an end host and a router. Denote the nodes in the network by {*N*_1_, *N*_2_,…, *N*_*a*_}. If node *N*_*i*_ is within the communication area of *N*_*k*_, then we say *N*_*i*_ is a neighbor of *N*_*k*_. Without loss of generality, we assume that wireless channels are symmetric; that is, whenever a node *N*_*i*_ is a neighbor of *N*_*k*_, *N*_*k*_ is also a neighbor of *N*_*i*_. Just as in previous work [3, 4], we assume the channel between any two neighboring nodes to be reciprocal. (This assumption implies that our work is most suitable for a homogeneous network. If the network is heterogeneous, then our work needs to be modified before it can be applied.) Denote the channel from *N*_*i*_ to *N*_*k*_ by *h*_*ik*_(*t*), and denote the channel from *N*_*k*_ to *N*_*i*_ by *h*_*ki*_(*t*). Then the channel reciprocity indicates that *h*_*ik*_(*t*) = *h*_*ki*_(*t*) for any time *t*.

We use the phase characteristics of both the initial signals and the channel as a random source to extract the shared secret key from. (Note that using the channel phase characteristics as a source of randomness is a feasible approach, which has been adopted in existing work, e.g., [34]. A possible way to implement this can be found in [35].) From the channel reciprocity, we know that within the channel coherence time, the channel between two nodes can be assumed to be invariant. We divide the channel coherence time to equal time slots: *T*_1_, *T*_2_,…, *T*_*d*_. Let the length of each time slot be *TS*, and denote the coherence time of the channel by *CT*. Let *d* = ⌊*CT*/*TS*⌋.

During one time slot *T*_*k*_, when *N*_*i*_ sends the initial signal to *N*_*j*_, we denote the signal sent from *N*_*i*_ by *s*_*i*_(*t*). *s*_*i*_(*t*) has the following representation:

(1) $$\begin{array}{c} s_{i}\left(t\right)=C_{i}\left(t\right)e^{j(\omega_{c}(t-t_{0})+\phi (t))}. \end{array}$$

In (1), *C*_*i*_(*t*) is the amplitude of *s*_*i*_(*t*). *ω*_*c*_ and *ϕ*(*t*) are the center frequency and the initial phase of *s*_*i*_(*t*), respectively. We emphasize that it is *feasible* to send a signal with a given phase *ϕ*(*t*)—in fact, some existing schemes like [34] already include such operations. In order to implement such an operation, one can use analog-to-digital converters [35].

*Definition of Adversaries.*  In this paper, we consider three different kinds of adversaries: *internal eavesdropper*, *external eavesdropper*, and *MITM adversary*. Here both internal eavesdroppers and external eavesdroppers refer to *passive* adversaries that eavesdrop messages and attempt to figure out the established key. The difference between these two types of adversaries is that an internal eavesdropper is an intermediate node in a path selected for transmitting messages for key agreement, while an external eavesdropper is not an intermediate node in any such path. Unlike these two types of passive adversaries, an MITM adversary is an *active* adversary who controls one or more node in a path selected for transmitting messages for key agreement and carries out an MITM attack. A little more formally, we have the following definitions.

**Definition 1**

A multihop key agreement scheme is secure against a set of external eavesdroppers if, assuming all involved nodes follow the protocol faithfully, all signals overheard by this set of eavesdroppers are statistically independent from the final key generated by this scheme.

**Definition 2**

A multihop key agreement scheme is secure against a set of internal eavesdropper if, assuming all involved nodes follow the protocol faithfully, all packets received by this set of eavesdroppers, together with all signals overheard by this set of eavesdropper, are statistically independent from the final key generated by this scheme.

**Definition 3**

A multihop key agreement scheme is secure against a set of MITM adversaries if, assuming all involved nodes except this set of MITM adversaries follow the protocol faithfully, the final keys different nodes obtain are consistent; furthermore, all packets received by this set of MITM adversaries, together with all signals overheard by this set of adversary, are statistically indepedent from the final key generated by this scheme.

## 4. The Basic Multihop Key Agreement Scheme

 In this section, we propose a basic multihop key agreement scheme. The basic scheme is built on one selected path between the two nodes that want to agree on a secret key. It is secure against any external eavesdroppers as long as those eavesdroppers are more than half a wavelength away from all the nodes in the selected path.

### 4.1. Scheme Outline

 The basic idea of this multihop key agreement scheme is to use both the channel phase characteristics of the selected path and the randomly selected initial phases to extract common secrets (i.e., secrets known only to *A* and *B*). By using quantization, these common secrets are quantized into common secret bits. After that, information reconciliation and privacy amplification are used [36–38] on the common secret bits, so that a secret key can be generated. When the external eavesdroppers are more than half a wavelength away, they will experience channels that are independent of the channels in the selected path [3, 4].

In order to have *k* common secret bits, the two parties (denoted by *A* and *B*) need to interact with each other for ⌈*k*/*q*⌉ rounds, assuming in each round that they can get *q* bits from quantization. In each round, *A* picks a random phase value, and sends an initial signal with this initial phase value to *B* using the selected path. Each intermediate node in this path estimates the phase of the signal received from its antecedent node and sends a new signal with this estimated phase to its subsequent node. Note that *A* is the first node in the path, and *B* is the last node in the path. Hence, *A* has a subsequent node only, and *B* has an antecedent node only. After *B* receives the signal from its antecedent node, it picks a random phase value and sends an initial signal with this initial phase value back to *A*, along the reverse path. Each intermediate node estimates the phase of the signal received from its subsequent node, and sends a new signal with the estimated phase to its antecedent node. Finally *B* (resp., *A*) estimates the phase of the signal received from its antecedent (resp., subsequent) node and adds the estimated phase with its randomly generated initial phase. The sums generated by *A* and *B* both reflect characteristics of all the channels in the path and the random initial phase values picked by *A* and *B*. In order to make sure that they are highly correlated, each round is completed within the channel coherence time. The random initial phase values picked by *A* and *B* are sources of randomness of the extracted common secrets.

After extracting common secrets from the channels and the random initial phase values, *A* and *B* perform independent quantization on these secrets and get common secret bits. The discrepancies between common secret bits of *A* and *B* are corrected by information reconciliation. The lost entropy of performing the information reconciliation is reduced by privacy amplification. In the following, we give detailed descriptions of these steps. After that, we give analysis of the basic scheme.

### 4.2. Common Secret Extraction

**Figure 1:**
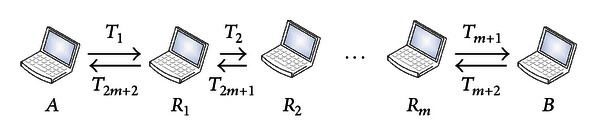
Illustration of signal transmission in one round.

The common secret extraction consists of ⌈*k*/*q*⌉ rounds, and each round contains (2*m* + 2) time slots.  Figure 1 illustrates the signal transmission involved in one round. 

In the following, we describe steps involved in one round.

(1) In the time slot *T*_1_ = [0,0 + *TS*], *A* sends the initial signal *s*_*a*_(*t*) with phase *ϕ*_1_ to *R*_1_, where the value of *ϕ*_1_ is randomly picked by *A* from [0,2*π*) (and thus *known to A*). Without loss of generality, we assume that *s*_*a*_(*t*) has a unit power level. Denote the signal received at *R*_1_ by *r*_*A*,*R*_1__. Then we get that *r*_*A*,*R*_1__(*t*) = *α*_*A*,*R*_1__(*t*)*e* ^*j*(*ω*_*c*_*t*+*ϕ*_*A*,*R*_1__)^ + *n*_*R*_1__(*t*), where *α*_*A*,*R*_1__(*t*) and *ϕ*_*A*,*R*_1__ denote the amplitude and phase of the signal received from *A*, and *n*_*R*_1__(*t*) denotes the receiver noise at *R*_1_.
(2) The phase of *r*_*A*,*R*_1__(*t*) is *ϕ*_*A*,*R*_1__ = *ϕ*_1_ + *ψ*_*A*,*R*_1__, in which *ψ*_*A*,*R*_1__ denotes the phase offset of the channel between *A* and *R*_1_. *R*_1_ computes the estimate of *ϕ*_*A*,*R*_1__, which we denote by ${\hat{\phi }}_{A,R_{1}}$. After that in *T*_2_, *R*_1_ sends a unit signal to *R*_2_ whose phase is tuned to ${\hat{\phi }}_{A,R_{1}}$.
(3) For *i* = 2,3,…, *m* − 1, in the time slot *T*_*i*+1_, *R*_*i*_ computes the phase estimate of the signal received from *R*_*i*−1_ and sends a new unit signal with this phase estimate to *R*_*i*+1_. In *T*_*m*+1_, *R*_*m*_ sends the signal $e^{j(w_{c}(t-m\cdot TS)+{\hat{\phi }}_{A,R_{m}})}$ to *B*.
(4) In the time slot *T*_*m*+2_, *B* sends the initial signal *s*_*b*_(*t*) with phase *ϕ*_2_ to *R*_*m*_, where *s*_*b*_(*t*) also has a unit power level, and *ϕ*_2_ is picked randomly by *B* from [0,2*π*) (and thus *known to B*). Denote the signal received at *R*_*m*_ by *r*_*B*,*R*_*m*__. Then *r*_*B*,*R*_*m*__(*t*) = *α*_*B*,*R*_*m*__(*t*)*e* ^*j*(*ω*_*c*_(*t*−(*m*+1)·*TS*)+*ϕ*_*B*,*R*_*m*__)^ + *n*_*R*_*m*__(*t*). The phase of *r*_*B*,*R*_*m*__(*t*) is *ϕ*_*B*,*R*_*m*__ = *ϕ*_2_ + *ψ*_*B*,*R*_*m*__, in which *ψ*_*B*,*R*_*m*__ denotes the phase of the channel between *B* and *R*_*m*_.
(5) For *i* = *m* + 3, *m* + 4,…, 2*m* + 1, in *T*_*i*_,  *R*_2*m*+3−*i*_ sends the signal $e^{j(w_{c}(t-(i-1)\cdot TS)+{\hat{\phi }}_{B,R_{2m+3-i}})}$ to *R*_2*m*+2−*i*_. In *T*_2*m*+2_, *R*_1_ sends the signal $e^{j(w_{c}(t-(2m+1)\cdot TS)+{\hat{\phi }}_{B,R_{1}})}$ to *A*.
(6) From the previous steps *B* receives *r*_*R*_*m*_,*B*_, and *A* receives *r*_*R*_1_,*A*_. It is easy to see that
  (2) $$\begin{array}{c} r_{R_{m},B}\left(t\right)=\alpha_{R_{m},B}\left(t\right)e^{j(w_{c}(t-m\cdot TS)+\phi_{A,B,1})}+n_{B}\left(t\right),\\ r_{R_{1},A}\left(t\right)=\alpha_{R_{1},A}\left(t\right)e^{j(w_{c}(t-(2m+1)\cdot TS)+\phi_{B,A,1})}+n_{A}\left(t\right), \end{array}$$
 where *ϕ*_*A*,*B*,1_ and *ϕ*_*B*,*A*,1_ denote the signal phases of *r*_*R*_*m*_,*B*_ and *r*_*R*_1_,*A*_, respectively. *B* computes $I_{B}=({\hat{\phi }}_{A,B,1}+\phi_{2})mod2\pi$, and *A* computes $I_{A}=({\hat{\phi }}_{B,A,1}+\phi_{1})mod2\pi$. From *I*_*B*_ and *I*_*A*_, *B* and *A* extract common secret bits.

We denote such a round by Round(*A*, *B*, *m*). Apparently Round(*A*, *B*, *m*) needs to take (2*m* + 2) time slots.

From the previous protocol process, we can get that $I_{B}=({\hat{\phi }}_{A,B,1}+\phi_{2})mod2\pi =\{est(\phi_{1}+\psi_{A,R_{1}}+\sum_{i=1}^{m-1}\psi_{R_{i},R_{i+1}}+\psi_{R_{m},B})+\phi_{2}\}mod2\pi$ and $I_{A}=({\hat{\phi }}_{B,A,1}+\phi_{1})mod2\pi =\{est(\phi_{2}+\psi_{B,R_{m}}+\sum_{i=1}^{m-1}\psi_{R_{i+1},R_{i}}+\psi_{R_{1},A})+\phi_{1}\}mod2\pi$. From the channel reciprocity, *I*_*B*_ and *I*_*A*_ are highly correlated if the measurements are within the channel coherence time. Hereafter, suppose that *A* and *B* carry out *z* rounds of Round(*A*, *B*, *m*), and denote the extracted secret vectors by [*I*_*A*,1_, *I*_*A*,2_,…, *I*_*A*,*z*_] and [*I*_*B*,1_, *I*_*B*,2_,…, *I*_*B*,*z*_], respectively.

### 4.3. Quantization

 After *z* rounds of common secret extraction, *A* has got the secret vector [*I*_*A*,1_, *I*_*A*,2_,…, *I*_*A*,*z*_], and *B* has got the secret vector [*I*_*B*,1_, *I*_*B*,2_,…, *I*_*B*,*z*_]. For *Z* ∈ {*A*, *B*}  and  *k* = 1,2,…, *z*,  *I*_*Z*,*k*_ is in the range of [0,2*π*). Now *A* and *B* quantize each value in their vectors into common secret bits. Specifically, we divide the interval [0,2*π*) into *q* equal subintervals. Denote these subintervals by [0, 2*π*/*q*), [2*π*/*q*, 4*π*/*q*),…, [2(*q* − 1)*π*/*q*, 2*π*). We quantize each subinterval into *log*_2_(*q*) bits using the Gray code [39]. By using Gray code, adjacent subintervals have only one bit discrepancy after quantization, which reduces the number of bit errors caused by estimation errors.

Denote the length of the targeted secret key by *k*. In order to generate the key, *A* and *B* need to interact with each other for at least ⌈*k*/*q*⌉ rounds.

### 4.4. Information Reconciliation and Privacy Amplification

 Because there exist noises and interferences at the receivers, *A* and *B* can get discrepancies at some common secret bits. They can achieve secret bits reconciliation by transmitting error correcting information through a public channel, which is called information reconciliation [40, 41]. We use the classic Cascade protocol [40] to perform reconciliation between the extracted secret bits. For completeness we briefly review the Cascade protocol.

Denote the two secret bit strings at *A* and *B* by *BS*_*A*_ and *BS*_*B*_. In the Cascade protocol, each of the two bit strings are divided into disjoint blocks. One party sends the parity values of all the blocks to the other party. If an odd number of errors are found within any block, *A* and *B* perform an interactive binary error search on that block, until one bit error is corrected. The Cascade protocol consists of several rounds, depending on the rate of bit discrepancies between *BS*_*A*_ and *BS*_*B*_. If in the *k*th (*k* ≥ 2) round, one error is corrected at the *i*th bit, and then any other block that contains the *i*th bit also contain an odd number of errors, which need to be corrected subsequently. Only minimal information gets leaked out if the number of rounds and the block size are selected appropriately.

After the information reconciliation, privacy amplification [36–38] is used to reduce the side information leaked during information reconciliation. We use the following 2-universal hash family [4]:

(3) $$\begin{array}{c} g_{a,b}\left(x\right)=\left(ax+b\right)mod\;p_{M},\\ h_{a,b}\left(x\right)=g_{a,b}\left(x\right)mod\;m,\;x\in \left\{1,2,\ldots ,M\right\},\\ a\in \left[1,p_{M}-1\right],\;\;b\in \left[0,p_{M}-1\right], \end{array}$$

where *p*_*M*_ is a prime number that satisfies *p*_*M*_ > *M*. This 2-universal hash family consists of all the functions *h* that map from {1,2,…, *M*} to {0,1}^*m*^. One party randomly selects *a* and *b* and sends them to the other party. We divide the secret bits after reconciliation into blocks of *log*_2_(*M*) bits, and *m* is decided based on the required secret key length.

After these two processes, the generated keys at *A* and *B* are cryptographic secure keys. *A* and *B* can use the generated key for secret communications.

### 4.5. Security Analysis of the Basic Scheme

 In this section, we present a security analysis of the basic scheme. Firstly we argue that the basic scheme is secure against any external eavesdroppers that are more than half a wavelength away from all the nodes in the selected path. Secondly we show that threats from internal adversaries can affect the security of the scheme. Finally we show that MITM attack is possible in the basic scheme. (Recall that internal eavesdroppers, external eavesdroppers, and MITM adversary are defined at the end of Section 3.)

#### 4.5.1. Security against Any External Eavesdropper

 If all the external eavesdroppers are more than half a wavelength away from all the nodes in the selected path, then their experienced channels are independent of channels between nodes in the selected path.

**Figure 2:**
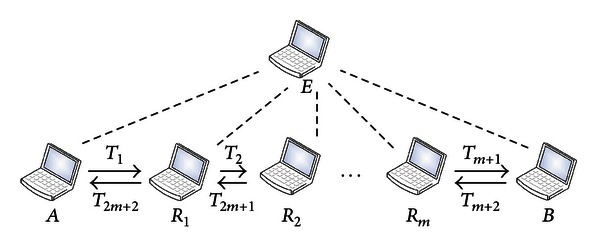
Illustration of one external eavesdropper in the basic scheme.

In the following we analyze the security of the basic scheme when there exists only one external eavesdropper. The analysis can be similarly extended to the case in which there are more than one eavesdroppers. In Figure 2, denote the eavesdropper by *E*. From Round(*A*, *B*, *m*), *E* gets the following estimated phases from its received signals:

(4) $$\begin{array}{c} est\left(\phi_{1}+\psi_{A,E}\right)\\ est\left(\phi_{1}+\psi_{A,R_{1}}+\sum_{i=1}^{k-1}\psi_{R_{i},R_{i+1}}+\psi_{R_{k},E}\right),\;k\in \left[1,m\right]\\ est\left(\phi_{2}+\psi_{B,E}\right)\\ est\left(\phi_{2}+\psi_{B,R_{m}}+\sum_{i=k}^{m-1}\psi_{R_{i+1},R_{i}}+\psi_{R_{k},E}\right),\;k\in \left[1,m\right]. \end{array}$$

In (4), *E* gets *est*(*ϕ*_1_ + *ψ*_*A*,*E*_) at *T*_1_ from *A* and gets *est*(*ϕ*_1_ + *ψ*_*A*,*R*_1__ + ∑_*i*=1_^*k*−1^ *ψ*_*R*_*i*_,*R*_*i*+1__ + *ψ*_*R*_*k*_,*E*_) at *T*_*k*+1_ from *R*_*k*_,  *k* ∈ [1, *m*]. On the other hand, *E* gets *est*(*ϕ*_2_ + *ψ*_*B*,*E*_) at *T*_*m*+2_ from *B* and gets *est*(*ϕ*_2_ + *ψ*_*B*,*R*_*m*__ + ∑_*i*=*k*_^*m*−1^ *ψ*_*R*_*i*+1_,*R*_*i*__ + *ψ*_*R*_*k*_,*E*_) at *T*_*m*+2+*k*_ from *R*_*m*+1−*k*_,  *k* ∈ [1, *m*].

Because *ϕ*_1_ and *ϕ*_2_ are randomly selected by *A* and *B*, respectively, these estimated phases are also random. Because *ψ*_*A*,*E*_ is independent of *ψ*_*A*,*R*_1__, *E* cannot get any knowledge of (*ϕ*_1_ + *ψ*_*A*,*R*_1__) from *est*(*ϕ*_1_ + *ψ*_*A*,*E*_). Similarly, *E* cannot get any knowledge of (*ϕ*_1_ + *ψ*_*A*,*R*_1__ + ∑_*i*=1_^*k*−1^ *ψ*_*R*_*i*_,*R*_*i*+1__ + *ψ*_*R*_*k*_,*B*_),  *k* ∈ [1, *m*] from *est*(*ϕ*_1_ + *ψ*_*A*,*R*_1__ + ∑_*i*=1_^*k*−1^ *ψ*_*R*_*i*_,*R*_*i*+1__ + *ψ*_*R*_*k*_,*E*_),  *k* ∈ [1, *m*]. Finally, during the channel coherence time, no probe signals are transmitted between the nodes in the selected path, so *ψ*_*A*,*E*_, *ψ*_*R*_*k*_,*E*_, *k* ∈ [1, *m*] and *ψ*_*B*,*E*_ are unknown to *E*. Therefore, from these estimated phase values, *E* gets no knowledge of the extracted secrets at *A* or *B*.

We stress that it is realistic to assume that the external eavesdroppers are at least half a wavelength away. When the carrier frequency is 2.437 GHz (one of the frequency band of 802.11 b), the wavelength of the carrier is (3 · 10^8^ m/s)/(2.437 · 10^9^ Hz) ≈ 0.12 m. Half a wavelength is only about 6 centimeters. Within such a distance, it is hard for an eavesdropper to avoid being detected.

#### 4.5.2. Threats of Internal Adversaries

 In the basic scheme, each of the internal nodes can get the complete knowledge of the extracted secrets at *A* and *B*. If one of them is corrupted, then the scheme is not secure. For example, if *R*_*k*_ is corrupted, based on its received signals from *R*_*k*−1_ and *R*_*k*+1_, it gets ${\hat{\phi }}_{A,R_{k}}=est(\phi_{1}+\psi_{A,R_{1}}+\sum_{i=1}^{k-1}\psi_{R_{i},R_{i+1}})$ and ${\hat{\phi }}_{B,R_{k}}=est(\phi_{2}+\sum_{i=k}^{m-1}\psi_{R_{i+1},R_{i}}+\psi_{B,R_{m}})$. By adding up these two values, *R*_*k*_ gets an estimate, which is highly correlated to both *I*_*B*_ and *I*_*A*_. Therefore, if one of the intermediate nodes is corrupted, the basic scheme is not secure.

#### 4.5.3. MITM Attack

 Because there are *m* intermediate nodes between *A* and *B*, any of them can carry out an MITM attack. Suppose that *R*_*k*_ intends to carry out an MITM attack and establish two different keys with *A* and *B*, respectively. Specifically, *R*_*k*_ agrees on one key with *A*, based on the subpath *A* → *R*_1_ → ⋯→*R*_*k*_; *R*_*k*_ agrees on another key with *B*, based on the other subpath *R*_*k*_ → *R*_*k*+1_ → ⋯→*B*. The MITM attack consists of the following steps:

1. In each round, *R*_*k*_ performs the following steps:
  - When *R*_*k*_ receives the signal *r*_*R*_*k*−1_,*R*_*k*__ = *α*_*R*_*k*−1_,*R*_*k*__(*t*)*e* ^*j*(*w*_*c*_(*t*−(*k*−1)·*TS*)+*ϕ*_*A*,*R*_*k*__)^ from *R*_*k*−1_, it picks a random value *ϕ*_*k*,1_ ∈ [0,2*π*) and sends *s*_*k*,1_(*t*) = *e* ^*j*(*w*_*c*_(*t*−*k*·*TS*)+*ϕ*_*k*,1_)^ to *R*_*k*+1_.
  - When *R*_*k*_ receives the signal *r*_*R*_*k*+1_,*R*_*k*__ = *α*_*R*_*k*+1_,*R*_*k*__(*t*)*e* ^*j*(*w*_*c*_(*t*−(2*m*+1−*k*)·*TS*)+*ϕ*_*B*,*R*_*k*__)^ from *R*_*k*+1_, it picks a random value *ϕ*_*k*,2_ ∈ [0,2*π*) and sends *s*_*k*,2_(*t*) = *e* ^*j*(*w*_*c*_(*t*−(2*m*+2−*k*)·*TS*)+*ϕ*_*k*,2_)^ to *R*_*k*−1_.
  - *R*_*k*_ computes the estimates of *ϕ*_*A*,*R*_*k*__ and *ϕ*_*B*,*R*_*k*__. Denote these two estimates by ${\hat{\phi }}_{A,R_{k}}$ and ${\hat{\phi }}_{B,R_{k}}$, respectively.
  - *R*_*k*_ computes $I_{k,B}={\hat{\phi }}_{B,R_{k}}+\phi_{k,1}$ and $I_{k,A}={\hat{\phi }}_{A,R_{k}}+\phi_{k,2}$. *R*_*k*_ then quantizes *I*_*k*,*B*_ and *I*_*k*,*A*_ to generate secret bit strings *Q*_*k*,*B*_ and *Q*_*k*,*A*_. Denote the length of *Q*_*k*,*B*_ and *Q*_*k*,*A*_ by *q* bits.
2. After *z* rounds, *R*_*k*_ gets *ST*_*k*,*B*_ = *Q*_*k*,*B*,1_||*Q*_*k*,*B*,2_||⋯||*Q*_*k*,*B*,*z*_ and *ST*_*k*,*A*_ = *Q*_*k*,*A*,1_||*Q*_*k*,*A*,2_||⋯||*Q*_*k*,*A*,*z*_, in which || denotes the string concatenation operation. Both *ST*_*k*,*B*_ and *ST*_*k*,*A*_ have a length of (*z* · *q*) bits. *R*_*k*_ uses *ST*_*k*,*B*_ to agree on a secret key KEY_*k*,*B*_ with *B*, and uses *ST*_*k*,*A*_ to agree on a secret key KEY_*k*,*A*_ with *A*.

From the attack process we can see that $I_{k,B}={\hat{\phi }}_{B,R_{k}}+\phi_{k,1}=est(\phi_{2}+\psi_{B,R_{m}}+\sum_{i=k}^{m-1}\psi_{R_{i+1},R_{i}})+\phi_{k,1}$, and *I*_*B*_ = *est*(*ϕ*_*k*,1_ + ∑_*i*=*k*_^*m*−1^ *ψ*_*R*_*i*_,*R*_*i*+1__ + *ψ*_*R*_*m*_,*B*_) + *ϕ*_2_. Both *I*_*k*,*B*_ and *I*_*B*_ can be viewed as estimates of *ϕ*_*k*,1_ + ∑_*i*=*k*_^*m*−1^ *ψ*_*R*_*i*_,*R*_*i*+1__ + *ψ*_*R*_*m*_,*B*_ + *ϕ*_2_. By using follow-up quantization, information reconciliation and privacy amplification techniques, *R*_*k*_ and *B* can agree on a secret key KEY_*k*,*B*_. Similarly, both *I*_*k*,*A*_ and *I*_*A*_ can be viewed as estimates of *ϕ*_*k*,2_ + ∑_*i*=1_^*k*−1^ *ψ*_*R*_*i*_,*R*_*i*+1__ + *ψ*_*A*,*R*_1__ + *ϕ*_1_. So *R*_*k*_ and *A* can also agree on a secret key KEY_*k*,*A*_. In this way, *R*_*k*_ carries out the MITM attack successfully.

### 4.6. Possible Reduction of Estimation Errors

 Given the basic scheme we have designed, there are possible ways to reduce the estimation errors. For instance, the intermediate nodes between *A* and *B* may append fix phase delay on forward and backward paths; that is, let Ψ_*R*_*i*_,*R*_*i*+1__ = Ψ_*R*_*i*+1_,*R*_*i*__. This would not reduce secrecy because *ϕ*_1_ and *ϕ*_2_ are random and unknown to the intermediate nodes.

## 5. The Improved Multihop Key Agreement

 Because the basic scheme suffers from threats of internal adversaries and the MITM attack, in this section, we propose an improved multihop key agreement scheme.

### 5.1. Scheme Outline

 In the improved multihop key agreement scheme, we assume that the network is biconnected. Therefore, between any pair of nodes, we can find at least two disjoint paths. The basic scheme suffers from threats from internal adversaries and the MITM attack because the signals are only transmitted in one path. Any node in that path can get knowledge of the extracted common secret bits and can perform the MITM attack. We design the improved multihop key agreement scheme to make it impossible for nodes in one path to get knowledge of the secret key or control it.

We emphasize that the previous goal of security is nontrivial to achieve. In particular, we consider a simple protocol, which we call SMPP hereafter. Assume that there are two disjoint paths Path_*A*_ and Path_*B*_ between *A* and *B*. SMPP starts by letting *A* and *B* generate key *K*_*A*_ over Path_*A*_ and key *K*_*B*_ over Path_*B*_. Then, *A* generates two random sequences *S*_*A*_ and *S*_*B*_, respectively, and sends *K*_*A*_ ⊕ *S*_*A*_ over Path_*B*_ to *B* and *K*_*B*_ ⊕ *S*_*B*_ over Path_*A*_ to *B*. Finally, *B* computes *S*_*A*_ by XORing his received value of *K*_*A*_ ⊕ *S*_*A*_ with *K*_*A*_; similarly, he computes *S*_*B*_. The final key agreed by *A* and *B* is the *S*_*A*_||*S*_*B*_.

Note that SMPP cannot really work against MITM attacks. For example, suppose that there is a node *N*_Adv_ controlled by the adversary in the middle of Path_*A*_. When *A* and *B* try to generate *K*_*A*_ over Path_*A*_, *N*_Adv_ launches an MITM attack and makes them disagree on the value of *K*_*A*_. (This is very easy in general, because *N*_Adv_ can simply play *B*'s role when talking to its neighbor on *A*'s side and play *A*'s role when talking to its neighbor on *B*'s side. In this way, *A* and *N*_Adv_ agree on one value of *K*_*A*_, while *N*_Adv_ and *B* agree on another value of *K*_*A*_.) Hence, *A* believes that the value of *K*_*A*_ is *K*_*A*_^*A*^, while *B* believes that the value of *K*_*A*_ is *K*_*A*_^*B*^. Both values (*K*_*A*_^*A*^ and *K*_*A*_^*B*^) are private against nodes in path *B*. Also suppose that all nodes in Path_*B*_ are honest and so *A* and *B* agree on the value of *K*_*B*_, which is private against nodes in Path_*A*_. Next, *A* generates *S*_*A*_ and *S*_*B*_ and sends *K*_*A*_^*A*^ ⊕ *S*_*A*_ over path *B* and *K*_*B*_ ⊕ *S*_*B*_ over path *A*. Assume that *N*_Adv_ does not tamper with these transmitted values. Therefore, *B* receives these values correctly. However, since *B* has a different belief about the value of *K*_*A*_, when *B* tries to recover the value of *S*_*A*_, he will get *K*_*A*_^*A*^ ⊕ *S*_*A*_ ⊕ *K*_*A*_^*B*^ instead of *S*_*A*_. In other words, *A* and *B* will disagree on the value of *S*_*A*_, which is part of the final key.

In order to achieve our goal of security, we use a better approach. We send the initial signals along two disjoint paths between *A* and *B*, perform estimation, and forwarding at intermediate nodes and add up the estimated phases of received signals from two paths at the two end nodes. In this way, the sum of phases contain not only the initial random values picked for phases, but also channel phase characteristics of both the two paths. Any adversaries within one single path can neither get the established secret key nor carry out a successful MITM attack.

In the improved multihop key agreement scheme, *A* and *B* jointly discover two disjoint paths between them. Denote the lengths of the two paths by *m* and *n*, respectively. After that, *A* and *B* carry out Round(*A*, *B*, *m*) along the first path and Round(*A*, *B*, *n*) along the second path. They interact with each other for sufficient rounds in order to get the targeted common secret bits. In each round, they add up extracted secrets from both rounds together. Finally, *A* and *B* perform quantization, information reconciliation and privacy amplification to get the secret key.

When performing the first step, existing node-disjoint routing discovery protocols [42, 43] can be used. In the improved scheme, we do not assume that there are any preloaded keys or public key infrastructures in the network. Secure routing protocols based on malicious node detection and trust based routing protocols [44–46] can meet this requirement. Using one of these protocols, *A* can find two disjoint paths to *B*. After that, *A* and *B* perform the rest of the multihop key agreement protocol by using the two paths.

### 5.2. The Improved Scheme—Detailed Description

**Figure 3:**
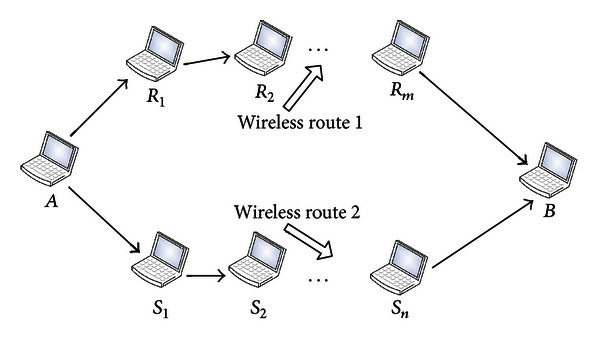
Disjoint routes between *A* and *B*.

 Denote the two disjoint paths between *A* and *B* by *A* → *R*_1_ → *R*_2_ → ⋯→*R*_*m*_ → *B* and *A* → *S*_1_ → *S*_2_ → ⋯→*S*_*n*_ → *B*, as shown in Figure 3.

The improved scheme consists of the following steps.

1. For *i* = 1 to *z*, *A* and *B* perform Round(*A*, *B*, *m*) along the first path and perform Round(*A*, *B*, *n*) along the second path. Without loss of generality, let *A* (resp., *B*) use the same initial phase *ϕ*_1,*i*_ (resp., *ϕ*_2,*i*_) for Round(*A*, *B*, *m*) and Round(*A*, *B*, *n*). We reset the starting time to 0 after each round. From Round(*A*, *B*, *m*), *A* and *B* get *I*_*A*,*i*_^(1)^ and *I*_*B*,*i*_^(1)^ as their extracted common secrets; from Round(*A*, *B*, *n*), *A* and *B* get *I*_*A*,*i*_^(2)^ and *I*_*B*,*i*_^(2)^ as their extracted common secrets. *A* and *B* get their final common secrets by computing *I*_*A*,*i*_ = *I*_*A*,*i*_^(1)^ + *I*_*A*,*i*_^(2)^ *mod*2*π* and *I*_*B*,*i*_ = *I*_*B*,*i*_^(1)^ + *I*_*B*,*i*_^(2)^ *mod*2*π*, respectively. Denote their extracted secret vectors by [*I*_*A*,1_, *I*_*A*,2_,…, *I*_*A*,*z*_] and [*I*_*B*,1_, *I*_*B*,2_,…, *I*_*B*,*z*_], respectively.
2. *A* quantizes each value in the vector [*I*_*A*,1_, *I*_*A*,2_,…, *I*_*A*,*z*_], and *B* quantizes each value in the vector [*I*_*B*,1_, *I*_*B*,2_,…, *I*_*B*,*z*_]. Denote their generated bit strings by *BS*_*A*_ and *BS*_*B*_, respectively.
3. *A* and *B* perform information reconciliation and privacy amplification on *BS*_*A*_ and *BS*_*B*_. After these two processes, they get the secret key.

### 5.3. Security Analysis

 In this section, we give a security analysis of the improved scheme. This security analysis is based on the assumption that all participating nodes are more than half a wavelength away from each other. Just as mentioned in Section 4.5.1, this is a reasonable assumption.

The security of the improved scheme is guaranteed against adversaries in a single path. Collusion attack from adversaries of both paths is not considered. In the following we first prove that the improved scheme is secure against any internal eavesdroppers in a single path. After that we prove that the improved scheme is secure against any MITM adversaries in a single path. (Recall that internal eavesdroppers and MITM adversary are defined at the end of Section 3.)

**Theorem 4**

Under the assumption that all nodes are more than half a wavelength away from each other, the improved multihop key agreement scheme is secure against any internal eavesdroppers in a single path.

**Proof**

In this proof we enumerate all the phase information that the routing nodes can extract and then point out that they cannot generate any useful information about *A* and *B*'s secrets.

In the following we consider the collected phase information at an intermediate node in one round. Because the extracted common secrets at each round are quantized separately, they cannot be used for getting knowledge of secrets of other rounds. Consider *R*_1_ in the first path *A* → *R*_1_ → *R*_2_ → ⋯→*R*_*m*_ → *B*. *R*_1_ receives signals from both *A* and *R*_2_. From the signals received from *A* and *R*_2_, *R*_1_ gets ${\hat{\phi }}_{A,R_{1}}=\phi_{1}+{\hat{\psi }}_{A,R_{1}}$ and ${\hat{\phi }}_{B,R_{1}}=est(\phi_{2}+\sum_{k=1}^{m-1}\psi_{R_{k+1},R_{k}}+\psi_{B,R_{m}})$, respectively. From these two phase estimates, *R*_1_ can only get the value of ${\hat{\phi }}_{A,R_{1}}+{\hat{\phi }}_{B,R_{1}}$. However, the secrets obtained by *A* and *B* also include the phase estimates through the other path *A* → *S*_1_ → *S*_2_ → ⋯→*S*_*n*_ → *B*. So we can see that *R*_1_ can get no information about the secrets.

For each intermediate node *R*_*k*_ in the first path, we enumerate its estimated phases as follows:

(5) $$\begin{array}{c} {\hat{\phi }}_{A,R_{k}}=est\left(\phi_{1}+\psi_{A,R_{1}}+\sum_{i=1}^{k-1}\psi_{R_{i},R_{i+1}}\right),\\ {\hat{\phi }}_{B,R_{k}}=est\left(\phi_{2}+\sum_{i=k}^{m-1}\psi_{R_{i+1},R_{i}}+\psi_{B,R_{m}}\right). \end{array}$$

Because all the *m* intermediate nodes are more than half a wavelength away from other nodes, they cannot get the phase information from the other path; that is, *ψ*_*A*,*S*_1__ + ∑_*k*=1_^*n*−1^ *ψ*_*S*_*k*_,*S*_*k*+1__ + *ψ*_*S*_*n*_,*B*_. No matter how many nodes in the first path combine their phase information, they cannot gain any knowledge about this value.

Therefore, we can see that the proposed protocol is secure against any internal eavesdroppers in one single path.

**Remark 5**

If an eavesdropper is not an intermediate node in either path, and he is more than half a wavelength away from all participating nodes, then he cannot gain any knowledge on the secret key either. This is similar to our analysis in Section 4.5.

**Theorem 6**

The improved multihop key agreement scheme is secure against any MITM adversaries in a single path.

**Proof**

Without loss of generality, suppose that *R*_*i*_ try to perform the MITM attack to *A* and *B*. The purpose of MITM attack is to establish two different keys with *A* and *B*, respectively, and after that to relay encrypted messages between them.

In Round(*A*, *B*, *m*), in *T*_*i*_, *R*_*i*_ receives the signal $r_{A,R_{i}}=\alpha_{R_{i-1},R_{i}}(t)e^{j(w_{c}(t-(i-1)\cdot TS)+{\hat{\phi }}_{A,R_{i-1}}+\psi_{R_{i-1},R_{i}})}+n_{R_{i}}(t)$ from *R*_*i*−1_. If *R*_*i*_ is an honest node, it will perform the phase estimation of the signal received from *R*_*i*−1_ and send the signal $e^{j(w_{c}(t-i\cdot TS)+{\hat{\phi }}_{A,R_{i}})}$ to *R*_*i*+1_. However, *R*_*i*_ wants to perform the MITM attack, so it generates *ϕ*_1_^*e*^ and sends a different signal *e* ^*j*(*w*_*c*_(*t*−*i*·*TS*)+*ϕ*_1_^*e*^)^ to *R*_*i*+1_. If all other nodes in the first path are honest, then the signal received by *B* should be

(6) $$\begin{array}{c} \alpha_{R_{m},B}\left(t\right)e^{j(w_{c}(t-m\cdot TS)+{\hat{\phi }}_{R_{i},R_{m}}^{e}+\psi_{R_{m},B})}+n_{B}\left(t\right). \end{array}$$

In (6), ${\hat{\phi }}_{R_{i},R_{m}}^{e}=est(\phi_{1}^{e}+\sum_{k=i}^{m-1}\psi_{R_{k},R_{k+1}})$.

On the other hand, when *R*_*i*_ receives $r_{R_{i+1},R_{i}}(t)=\alpha_{R_{i+1},R_{i}}(t)e^{j(w_{c}(t-(2m+2-i)\cdot TS)+{\hat{\phi }}_{B,R_{i+1}}+\psi_{R_{i+1},R_{i}})}+n_{R_{i}}(t)$ from *R*_*i*+1_ in *T*_2*m*+2−*i*_, *R*_*i*_ generates another phase *ϕ*_2_^*e*^ and sends *e* ^*j*(*w*_*c*_(*t*−(2*m*+3−*i*)·*TS*)+*ϕ*_2_^*e*^)^ to *R*_*i*−1_. If *R*_1_, *R*_2_,…,  and  *R*_*i*−1_ behave honestly, and then the signal *A* receives should be

(7) $$\begin{array}{c} \alpha_{R_{1},A}\left(t\right)e^{j(w_{c}(t-(2m+2)\cdot TS)+{\hat{\phi }}_{R_{i},R_{1}}^{e}+\psi_{R_{1},A})}+n_{B}\left(t\right). \end{array}$$

In (9),  ${\hat{\phi }}_{R_{i},R_{1}}^{e}=est(\phi_{2}^{e}+\sum_{k=1}^{i-1}\psi_{R_{k+1},R_{k}})$.

Now *B* can get his secret bits by quantizing *est*(*ϕ*_1_^*e*^ + ∑_*k*=*i*_^*m*−1^ *ψ*_*R*_*k*_,*R*_*k*+1__ + *ψ*_*R*_*m*_,*B*_) + *I*_*B*_^(2)^ + *ϕ*_2_. *A* can get its secret bits by quantizing *est*(*ϕ*_2_^*e*^ + ∑_*k*=1_^*i*−1^ *ψ*_*R*_*k*+1_,*R*_*k*__ + *ψ*_*R*_1_,*A*_) + *I*_*A*_^(2)^ + *ϕ*_1_. *R*_*i*_ has *est*(*ϕ*_2_ + *ψ*_*B*,*R*_*m*__ + ∑_*k*=*i*_^*m*−1^ *ψ*_*R*_*k*+1_,*R*_*k*__) + *ϕ*_1_^*e*^ and *est*(*ϕ*_1_ + *ψ*_*A*,*R*_1__ + ∑_*k*=1_^*i*−1^ *ψ*_*R*_*k*_,*R*_*k*+1__) + *ϕ*_2_^*e*^. However, *R*_*i*_ does not know *I*_*B*_^(2)^ and *I*_*A*_^(2)^ either, because *R*_*i*_ is more than half a wavelength from the other path.

From the previous analysis we know that *R*_*i*_ cannot agree on two different keys with *A* and *B*. Therefore, it cannot carry out MITM attack successfully. This analysis can be directly extended to the case that any number of intermediate nodes in the first path carry out MITM attacks collaboratively. Because their experienced channels are statistically independent of channels of the second path, they cannot gain any information of *I*_*B*_^(2)^ or *I*_*A*_^(2)^.

We conclude that the improved protocol is secure against any MITM adversaries in a single path.

**Remark 7**

If the adversary can place cheating nodes on two disjoint paths, there are straightforward ways to extend our protocol to achieve security. For example, we can consider using three disjoint paths between *A* and *B*. In general, in order to prevent cheating nodes on *k* disjoint paths, *A* and *B* can use *k* + 1 disjoint paths between them for key extraction, as long as there exist *k* + 1 disjoint paths between them. (If there are cheating nodes on all disjoint paths between *A* and *B*, then no solution is possible because these nodes can choose to simply block all communications between *A* and *B*.) This will lead to higher complexity of the protocol—so, there is a tradeoff between security and efficiency.

## 6. Performance Analysis

 As the improved protocol has more than just a pair of nodes, the estimation errors at each intermediate node will aggregate. In this section we present performance analysis of the improved protocol. We mainly focus on the agreement probability of *A* and *B*'s common secrets.

From the protocol description, we know that the ideal values of *I*_*A*_ and *I*_*B*_ are as follows:

(8) IA−=2ϕ2+ψB,Rm+∑i=2mψRi,Ri−1+ψR1,A+ψB,Sn+∑i=2nψSi,Si−1+ψS1,A+2ϕ1,IB−=2ϕ1+ψA,R1+∑i=1m−1ψRi,Ri+1+ψRm,B+ψA,S1+∑i=1n−1ψSi,Si+1+ψSn,B+2ϕ2.

From the channel reciprocity and the assumption that one protocol round is performed within the channel coherence time, we can see that $\overset{-}{I_{A}}=\overset{-}{I_{B}}$. We denote this value by $\overset{-}{I}$; that is, $\overset{-}{I}=\overset{-}{I_{A}}=\overset{-}{I_{B}}$. However, due to the estimation errors of the phase information, there may be discrepancies between *I*_*A*_ and *I*_*B*_. In the following we analyze the probability of *I*_*A*_ = *I*_*B*_ during one protocol round. We denote this probability by *P*_*r*_.

When one node transmits signals to another node, they use the same frequency, so that the receiver does not need to do frequency estimation. Without loss of generality, the noises at the receivers are independent Gaussian noises with zero mean and variance *σ* ^2^. The receiver samples the received signal and computes the phase estimate. When the sampling rate is high enough, the estimated phase is a Gaussian random variable whose variance is bounded by the Cramér-Rao bound [47].

From [47], when the signal frequency is known, the variance of the phase is bounded by

(9) $$\begin{array}{c} \sigma_{\hat{\theta }}^{2}\geq \frac{\sigma^{2}}{b_{0}^{2}N}. \end{array}$$

In (9), *b*_0_ is the amplitude of the received signal. From (9), we can see that the lower bound of the phase variance depends on the signal to noise ratio (SNR) and the sampling rate. When the SNR is higher, the phase variance can achieve a smaller lower bound. When the sampling rate is increased at the receiver, the lower bound can be further decreased. This is in accordance with the intuition that we should get more precise estimation given a higher SNR and sampling rate. In the following we use the Cramér-Rao bound for our analysis.

The estimation error at each node is modeled as a Gaussian noise, with the zero mean and standard deviation relying on the SNR and the sampling rate. Without loss of generality, we assume that the SNR and the sampling rate are all the same at all the participating nodes. From the protocol execution process, we know that the accumulated estimation error at the source or the destination is the sum of all the intermediate estimation errors. We can write *I*_*B*_ as

(10) $$\begin{array}{c} I_{B}=\overset{-}{I_{B}}+Z_{B}. \end{array}$$

*Z*_*B*_ represents the accumulated estimation error at *B*. According to the previous analysis, $Z_{B}\sim N(0,(m+n+2)\sigma_{\hat{\theta }}^{2})$. Because $\overset{-}{I_{B}}=\overset{-}{I}$,  $I_{B}\sim N(\overset{-}{I},(m+n+2)\sigma_{\hat{\theta }}^{2})$. For ease of analysis, let $\sigma_{I}^{2}=(m+n+2)\sigma_{\hat{\theta }}^{2}$. From the protocol execution process, we know that $I_{A}\sim N(\overset{-}{I},\sigma_{I}^{2})$. Because $\overset{-}{I}\in [0,2\pi )$, from the property of Gaussian distribution, the probability is much higher when *I*_*B*_ and *I*_*A*_ are close to $\overset{-}{I}$.

The probability *P*_*r*_ is a function of $\overset{-}{I}$. It can be computed using the following equation:

(11) Pr=∑i=0q−1P[IA∈[2πiq,2π(i+1)q),   IB∈[2πiq,2π(i+1)q)].

Because of the independent noise accumulations at *A* and *B*, we can get

(12) Pr=∑i=0q−1P[IA∈[2πiq,2π(i+1)q)]×P[IB∈[2πiq,2π(i+1)q)].

Denote the interval [2*πi*/*q*, 2*π*(*i* + 1)/*q*) by *Q*_*i*_. Let *P* ^*i*^(*A*, *B*) = *P*[*I*_*A*_ ∈ *Q*_*i*_]*P*[*I*_*B*_ ∈ *Q*_*i*_]. Then from the distribution function of Gaussian distribution, $P^{i}(A,B)=\int_{I_{A}\in Q_{i}}\left(1/\sqrt{2\pi }\sigma_{I}\right)e^{-{\left(I_{A}-\overset{-}{I}\right)}^{2}/2\sigma_{I}^{2}}\int_{I_{B}\in Q_{i}}\left(1/\sqrt{2\pi }\sigma_{I}\right)e^{-{\left(I_{B}-\overset{-}{I}\right)}^{2}/2\sigma_{I}^{2}}$. Because *I*_*A*_ and *I*_*B*_ have the same distributions, *P*_*r*_ can be computed by the following expressions:

(13) $$\begin{array}{c} P_{r}=\sum_{i=0}^{q-1}P^{i}\left(A,B\right),\\ P^{i}\left(A,B\right)={\left(\int_{I_{A}\in Q_{i}}\frac{1}{\sqrt{2\pi }\sigma_{I}}e^{-{\left(I_{A}-\overset{-}{I}\right)}^{2}/2\sigma_{I}^{2}}\right)}^{2}. \end{array}$$

From (13) we can see that *P*_*r*_ is the sum of the probability that *I*_*A*_ and *I*_*B*_ fall into the same quantization subinterval; that is, *P* ^*i*^(*A*, *B*),  *i* = 1,2,…, *q*. For each subinterval *Q*_*i*_, the magnitude of *P* ^*i*^(*A*, *B*) is affected by whether $\overset{-}{I}\in Q_{i}$. Suppose that $\overset{-}{I}\in Q_{i^{*}}$, and then *P* ^i*^(*A*, *B*) will be larger than any other *P* ^*i*^(*A*, *B*) for $\overset{-}{I}\notin Q_{i}$. This is because the Gaussian distribution function has a larger value when the variable value is closer to the mean (in this case, $\overset{-}{I}$). Therefore, *P*_*r*_ is dominated by *P* ^i*^(*A*, *B*), for $\overset{-}{I}\in Q_{i^{*}}$. On the other hand, *P* ^i*^(*A*, *B*) is affected by $\overset{-}{I}$'s position in *Q*_i*_. If $\overset{-}{I}$ is close to the center of *Q*_i*_, then *P* ^i*^(*A*, *B*) will be large; if $\overset{-}{I}$ is close to the end points of *Q*_i*_, then *P* ^i*^(*A*, *B*) will be small. This is because when $\overset{-}{I}$ is close to the end points, the probability that *I*_*A*_ and *I*_*B*_ fall into two adjacent subintervals increases. In addition, the standard deviation *σ*_*I*_ also has impact on *P* ^i*^(*A*, *B*). A smaller *σ*_*I*_ will result in a larger *P* ^i*^(*A*, *B*), because when *σ*_*I*_ is smaller, the probability of *I*_*A*_ or *I*_*B*_ being close to $\overset{-}{I}$ is larger.

## 7. Simulation Results

**Figure 4:**
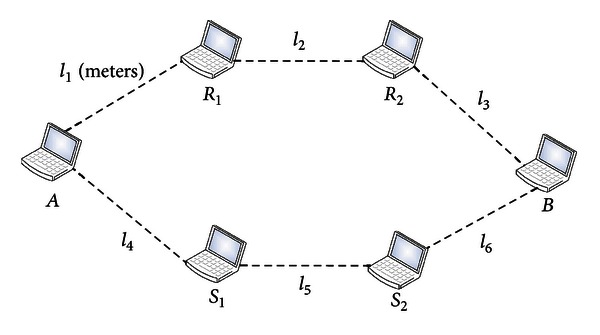
One common node distribution of the simulation.

**Figure 5:**
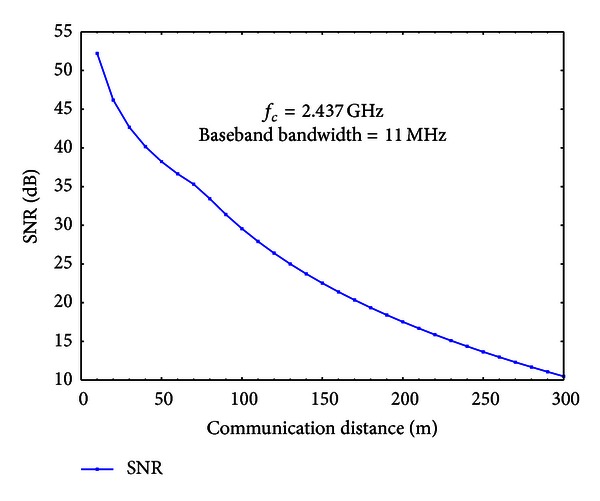
Average SNRs under different communication distances.

 In order to measure the performance of the proposed scheme, we simulate the proposed scheme using GlomoSim [7]. By using the PARSEC programming language [48], we write programs for the proposed scheme in the physical layer of GlomoSim protocol stack. We simulate the proposed scheme for different SNRs. Because the receiver SNR is affected mainly by distances between adjacent nodes, we select a set of communication distances, which is {10 m, 20 m, 30 m, 40 m, 50 m, 100 m, 150 m, 200 m, 250 m, 300 m}. For each communication distance (denote it by *l*), we randomly generate a geometric distribution of 6 nodes. The distance between any pair of adjacent nodes is randomly generated in [0.7*l*, 1.3*l*]. We denote these distances by {*l*_1_, *l*_2_, *l*_3_, *l*_4_, *l*_5_, *l*_6_}. Because we select 10 communication distances, we also generate 10 random distributions of nodes. One common node distribution for the simulation is shown in Figure 4. We measure average SNRs under different communication distances. The results are shown in Figure 5.

To best simulate the wireless communication environment in reality, we set the center carrier frequency to be 2.437 GHz and the baseband bandwidth to be 11 MHz. This is one of the standard carrier band of 802.11 b. According to Nyquist-Shannon sampling theorem, the sampling rate should be no less than 22 MHz. We choose the sampling rate to be 25 MHz, so that the estimation at the receiver is more accurate. *TS* is chosen to be 10 *μ*s. For the large scale signal propagation, we use the two-ray ground reflection model [49] which can be expressed by (14)

(14) $$\begin{array}{c} P_{r}\left(d\right)=P_{t}G_{t}G_{r}\frac{h_{t}^{2}h_{r}^{2}}{d^{4}}. \end{array}$$

In (14), *P*_*t*_ is the transmission power, and *P*_*r*_(*d*) is the received power at a distance *d* away from the transmission antenna. *G*_*t*_ and *G*_*r*_ are the antenna gains at the transmitter and the receiver, respectively; *h*_*t*_ and *h*_*r*_ are the antenna heights at the transmitter and the receiver, respectively; *d* is the distance between the transmitter and the receiver.

We use the Rayleigh distribution [49] for the small scale wireless fading model. Both the two-ray ground reflection model and the Rayleigh fading model are directly supported by the GlomoSim network simulator [7].

We measure the quantization agreement probability of *A* and *B* under different communication distances. We also measure the randomness of the secret key. In addition, we measure the key efficiency of the proposed scheme. The results are shown in Sections 7.1, 7.2, and 7.3.

### 7.1. Quantization Agreement Probability

 Under different communication distances, we measure quantization agreement probabilities and bit error rates (BERs) of the quantized common secret bits. For the quantization step, we choose *q* = 32. Therefore, the interval of [0,2*π*) is divided into 32 subintervals of equal length. We use the Gray code to encode the quantization indices, so that only one bit discrepancy is introduced for adjacent intervals.

**Figure 6:**
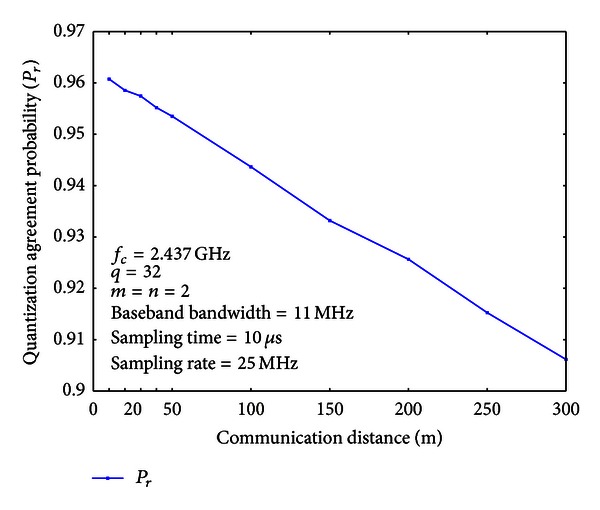
Quantization agreement probabilities under different communication distances.

**Figure 7:**
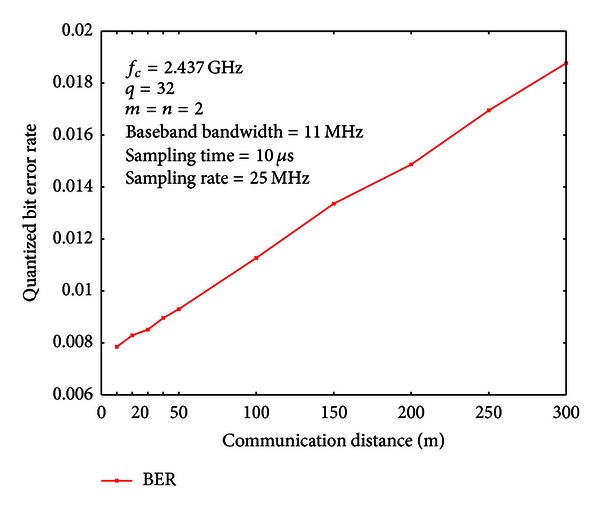
Bit error rates under different communication distances.

The results are shown in Figures 6 and 7, respectively. From Figures 6 and 7, we can see that when the communication distance is 50 m (approximately 38.23 dB SNR), the quantization agreement probability is 0.9535, and the BER is 0.0093. Even when the communication distance is increased to 300 m (approximately 10 dB SNR), the quantization agreement probability is still 0.906, and the BER is 0.019.

### 7.2. Randomness of the Generated Key

 We test the randomness of the generated key using the NIST randomness test suite [50]. We use the 8 tests in the NIST test suite to validate the randomness of one 1024-bit key. The results are shown in Table 1. From Table 1 we can see that the generated key passes all the 8 tests.

### 7.3. Key Efficiency

 In this section, we focus on measuring how long it takes in order to generate a 256-bit key. In order to generate a 256-bit key, *A* and *B* need to get more common secret bits, because the Cascade protocol causes entropy loss. We compute the lost entropy rate of Cascade protocol according to the theoretical results in [40]. After that we measure the key efficiency under different Cascade parameters.

**Figure 8:**
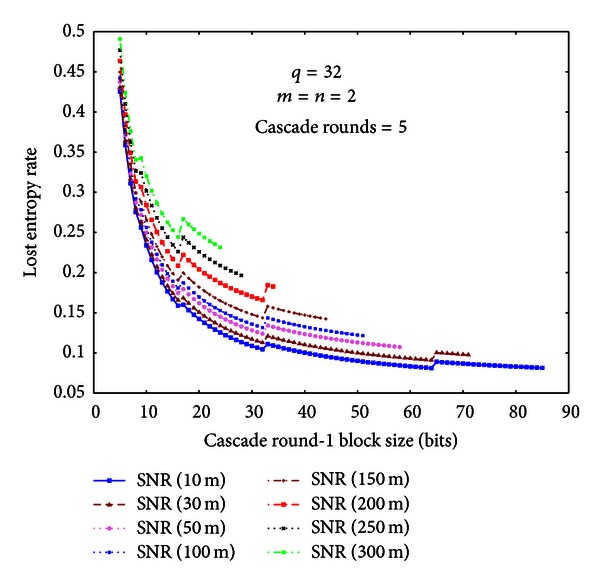
Lost entropy rates under different SNRs and Round-1 cascade blocks.

We have completely implemented the Cascade protocol and the privacy amplification method described in Section 4.4. We use the MIRACL library to implement the prime generation and large number arithmetics required for 2-universal hash family. We choose 4~5 rounds for the Cascade protocol, in order that the key agreement ratio is high. We compute the entropy loss rate when the Round-1 block size has different values. For each Round-(*i* + 1), its block size is two times the block size of Round-*i*. The results are shown in Figure 8.

As can be seen from Figure 8, when the Round-1 block of Cascade protocol increases, the lost entropy rate decreases. When the communication distance decreases, the lost entropy rate also decreases, because less bits need to be corrected. For example, when the communication distance is 50 m and the round-1 block size is 14, the lost entropy rate is 0.1925. Under such a lost entropy rate, in order to generate a 256-bit key, at least 317 common secret bits need to be collected. When the communication distance is 300 m and the round-1 block size is 10, the lost entropy rate is 0.3203. Under such a lost entropy rate, in order to generate a 256-bit key, at least 376 common secret bits need to be collected.

**Figure 9:**
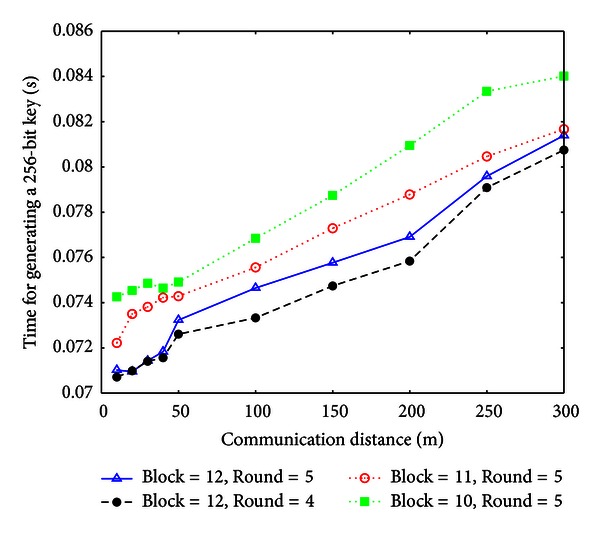
Efficiency of key generation under different communication distances. The measured time is for generating a 256-bit key.

Under the 10 distributions generated for different communication distances, we measure the efficiency of generating a 256-bit key using the multihop key agreement protocol. Different combinations of Cascade rounds and Round-1 block sizes are used. The simulation is run at a laptop with Intel Core2 CPU of 2.33 GHz and 2.0 GB memory. For each different setting, we run the key agreement scheme for 100 times and measure the average time. In all these executions, *A* and *B* achieve successful key agreement. The efficiency results are shown in Figure 9.

From Figure 9, we can see that when the Cascade Round-1 block size is decreased, the key efficiency is also decreased. This is because the block number is increased, which increases transferred bits in each round. Furthermore, when the number of Cascade rounds is decreased, the key efficiency is increased. Specifically, for the Cascade parameter (Block = 12, Round = 4), when the communication distance is 50 m, the time of generating a 256-bit key is 0.0726 seconds. At this speed, the proposed key agreement scheme can achieve 3.5 Kbps rate. Even when the communication distance is 300 m, the proposed scheme can still achieve 3.17 Kbps rate.

## 8. Conclusions and Discussions

 In this paper, we propose two key agreement schemes as a novel physical-layer technique in multihop wireless networks. The proposed key agreement schemes enable secret key generation between nodes in multihop wireless networks, even if they cannot communicate with each other directly. The proposed basic scheme is secure against external eavesdroppers. And the improved two-path-based scheme is secure against external eavesdroppers, as well as internal eavesdroppers and MITM adversaries in a single path. The proposed scheme can achieve high key efficiency under different communication distances among nodes. The secret key generated by the proposed scheme has very strong randomness. By properly selecting the protocol parameters, the proposed scheme can achieve high success ratio. The proposed scheme is suitable for establishing secret keys for multihop wireless networks.

It is worth noting that our paper has covered only key agreement for unicast communications between two nodes. Broadcast and multicast communications may require different protocols for key agreement. In particular, key agreement for broadcast communications in a wireless network is relatively easy if there are only passive eavesdroppers. A straightforward solution is to establish key agreement between neighbor nodes and then transmit a global key in encrypted form throughout the network. If some nodes in the network are dishonest, then leaking the final global key is unavoidable.

For multicast communications, this problem becomes the pretty challenging problem of group key agreement. Existing solutions such as Wang et al.'s [34] are suitable for this case, but further improvement in security and/or efficiency is also possible.

## Figures and Tables

**Table 1:** NIST statistical test results. To pass each test, the *P* value needs to be greater than 0.01.

Test	*P* value
Frequency	0.70766
Block frequency	0.936991
Runs	0.658522
Longest run of ones	0.871862
FFT	0.066457
Serial	0.815653, 0.586988
Approximate entropy	0.323517
Cumulative sums (forward)	0.745842
Cumulative sums (reverse)	0.745842
